# Unstable eigenmodes are possible drivers for cardiac arrhythmias

**DOI:** 10.1098/rsif.2011.0152

**Published:** 2011-05-13

**Authors:** Aslak Tveito, Glenn Lines, Ola Skavhaug, Mary M. Maleckar

**Affiliations:** 1 Simula Research Laboratory, Centre for Biomedical Computing, PO Box 134, Lysaker 1325, Norway; 2 Department of Informatics, University of Oslo, PO Box 1072, 0316 Oslo, Norway

**Keywords:** bioelectricity, cardiac arrhythmia mechanisms, eigenmode analysis

## Abstract

The well-organized contraction of each heartbeat is enabled by an electrical wave traversing and exciting the myocardium in a regular manner. Perturbations to this wave, referred to as arrhythmias, can lead to lethal fibrillation if not treated within minutes. One manner in which arrhythmias originate is an ill-fated interaction of the regular electrical signal controlling the heartbeat, the sinus wave, with an ectopic stimulus. It is not fully understood how and when ectopic waves are generated. Based on mathematical models, we show that ectopic beats can be characterized in terms of unstable eigenmodes of the resting state.

Cardiac arrhythmias are characterized by disturbances in the regular electrical signal that controls contraction of cardiac muscle. Arrhythmias decrease the ability of the heart to pump blood, and may degenerate into the disorganized, lethal electrical activity known as fibrillation. Decades of research have thus been devoted to understanding arrhythmic origins. It is well established that a fast (tachy)arrhythmia can be induced via the interaction of the normal cardiac action potential with an additional electrical stimulus [1–4]. Additional stimuli, arising inappropriately and without warning, are known as ectopic foci. While a young, healthy person is likely to experience about one ectopic beat per day [5], patients with compromised cardiac function may have as many as two ectopic beats per minute [6]. Despite the paramount importance of ectopic activity in the generation and maintenance of arrhythmias, its origins in cardiac disease have still not been completely characterized [7–9].

To date, tremendous insight into the mechanisms of arrhythmia has been afforded by mathematical models of cardiac electrophysiology [10,11]. A striking example is the concept of the phase singularity, long known in physics and mathematics, which underlies the induction of reentrant arrhythmias as outlined above [12,13]. Similarly, eigenmode analysis of mathematical models is commonly used in many fields of science and engineering in order to assess the stability of a physical state; we show here that such analysis is again applicable to cardiac arrhythmogenesis [14–16], and is able to explain the stochastic appearance of ectopic foci via destabilization of the resting electrophysiological state.

Box 1. The mathematical model under consideration can be written on the form1
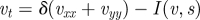
and2
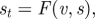
where *δ* is the electrical diffusion coefficient of the tissue, *v* is the transmembrane potential, *F* represents the electrochemical processes underpinning each action potential and *s* carries additional dynamical variables; a complete presentation is given in the electronic supplementary material. The system is equipped with an initial condition (*v*^0^, *s*^0^) and with no-flux boundary conditions. We also consider a set of perturbed initial conditions given by 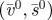, giving rise to the solution 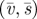. We are interested in analysing the behaviour of the difference between these solutions as given by 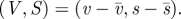 Up to linear terms, the difference is governed by3
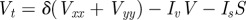
and4
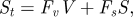
where *I*_*v*_ = ∂*I*(*v*^0^, *s*^0^)/∂*v*, and *I*_*s*_ = ∑_*i*_∂*I*(*v*^0^, *s*^0^)/∂*s*_*i*_; similar for *F*_*v*_ and *F*_*s*_. This system can be discretized in space on a computational grid and written in the form5
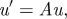
where *u* = *u*(*t*) is a vector containing grid values of *V* and *S*, and *A* is the associated system matrix [17,18]. Suppose that *λ* is an eigenvalue of *A* and *r* is the associated eigenvector; then a solution of the system is given by *e*^*λt*^*r*, and thus a perturbation containing the eigenvector *r* is unstable provided that the real part of *λ* is positive [19].

Conduction of the cardiac action potential in tissue can be described mathematically in the generic form presented in box 1. The aim of this report is to analyse how aberrant cardiac physiology, as may occur in post-injury remodelling, may influence the likelihood of generating an ectopic beat in simulated tissue. Considered are (i) the presence of electrically active, coupled fibroblasts which may arise as result of fibrosis [20] in the post-infarct heart, (ii) an increase in stretch-activated currents that may follow tissue dilatation associated with heart failure [21,22], and (iii) altered baseline repolarization currents, as seen in cardiac hypertrophy [23,24]. Alterations were incorporated into select membrane models (presented in their entirety in the electronic supplementary material). First considered is the simple, internally consistent, and computationally efficient cardiac action potential model developed by Krogh-Madsen *et al.* [25]. The atrial myocyte model of Maleckar *et al.* [26,27] is also included in the analysis to consider a biophysically based model grounded in human physiology.

All conditions (i–iii), as outlined above, result in a qualitatively comparable destabilization of the resting state of the tissue; analysis reveals that the dominant eigenvalues of the linearized system increase, changing sign from negative to positive. When an eigenvalue has a positive real part, perturbation of the cells' state by the associated eigenvector will result in instability (box 1). For the sake of brevity, representative disease conditions (i) and (ii) are presented below, while results following condition (iii) are presented in the electronic supplementary material.

Figure 1 shows the transmembrane potential following perturbation of a solution with eigenvectors of corresponding negative and positive eigenvalues. A square region in the myocardial sheet includes active, coupled fibroblasts. The solution is first perturbed by an eigenvector associated with a negative eigenvalue (left column), and the perturbation dies out rapidly (2 ms) after it is applied. However, perturbation via a primary eigenvector (right column) results in maintenance and growth of the perturbation. The final result is a full-blown activation: an ectopic focus.

**Figure 1.**
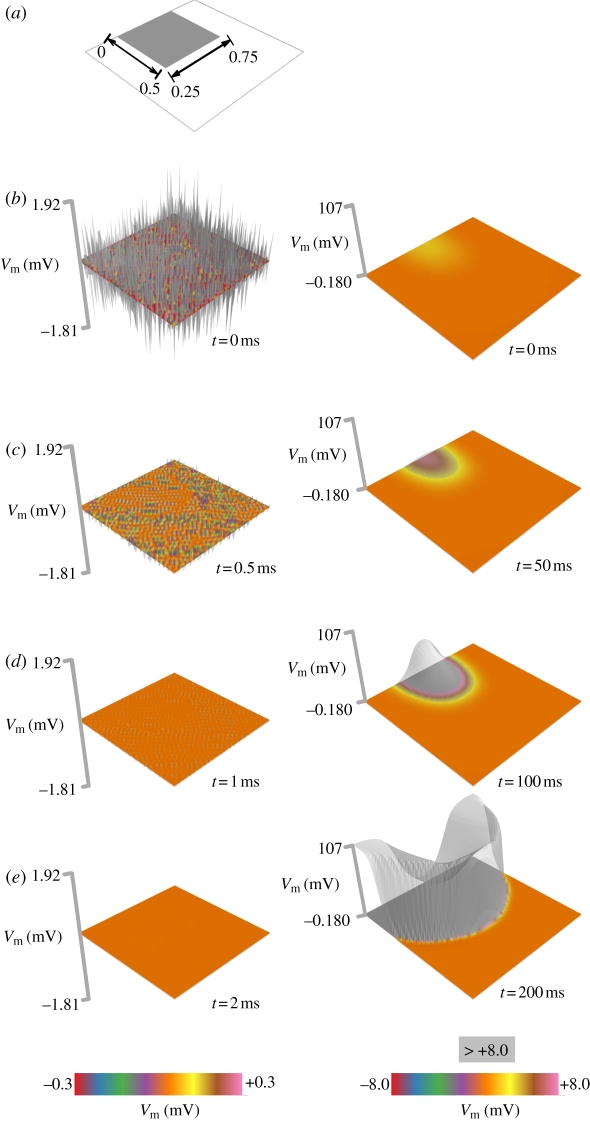
Evolution of stable and unstable perturbations in two-dimensional cardiac tissue incorporating fibroblasts. (*a*) The electrophysiology of the tissue substrate is modelled by the Krogh-Madsen model. The shaded square shows the region incorporating fibroblasts (*η* = 4, cf. the electronic supplementary material), as may be present during fibrotic remodelling. (*b*) Both stable (at left) and unstable (at right) perturbations at *t* = 0 ms. (*c*) Early evolution of the stable (at left, *t* = 0.5 ms) and unstable perturbations (at right, *t* = 1 ms). (*d*) Further evolution of the stable (at left, *t* = 1 ms) and unstable perturbations (at right, *t* = 50 ms). The latter has begun to grow in magnitude. (*e*) At *t* = 2 ms, the stable perturbation (at left) has completely died out, while by *t* = 200 ms, the unstable perturbation has induced a full-blown activation in the tissue: an ectopic beat.

The influence of stretch-activated currents (condition (i)) on the electrophysiological stability of myocardium is also examined by their inclusion in a square region of a myocardial sheet (figure 2). Accounting for the influence of stretch-activated currents alters the electrophysiological substrate; analysis reveals that the primary system eigenvalues change sign from negative to positive. Both stable (left column) and unstable (right column) perturbations are applied; the latter induces activity on the lateral border of the region containing stretch-activated currents. Further evolution reveals that the unstable perturbation has induced a propagating ectopic focus.

**Figure 2.**
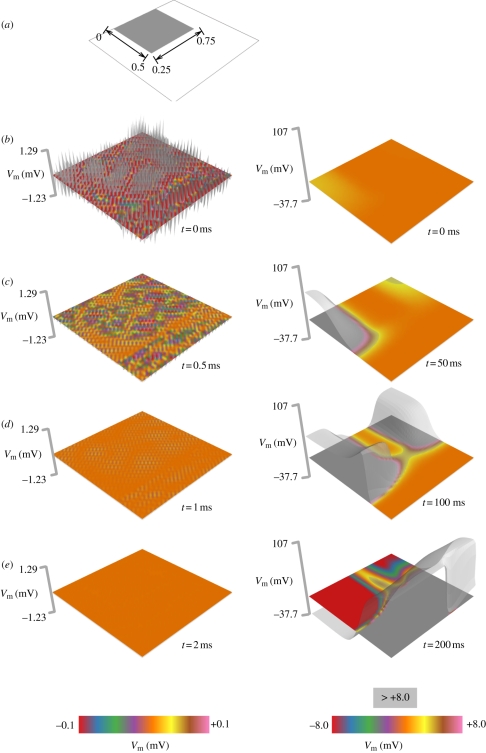
Evolution of stable and unstable perturbations in two-dimensional cardiac tissue incorporating stretch-activated currents. (*a*) The electrophysiology of the tissue substrate is modelled by the Krogh-Madsen model. The shaded square shows the region incorporating stretch-activated currents (*g*_sac_ = 0.5, cf. the electronic supplementary material), as may be present in dilated tissues during heart failure. (*b*) Both stable (at left) and unstable (at right) perturbations at *t* = 0 ms. (*c*) Early evolution of the stable (at left, *t* = 0.5 ms) and unstable perturbations (at right, *t* = 50 ms). The latter has induced activity on the lateral border of the region containing stretch-activated currents. (*d*) Further evolution of the stable (at left, *t* = 1 ms) and unstable perturbations (at right, *t* = 100 ms). (*e*) At *t* = 2 ms, the stable perturbation (at left) has completely died out, while by *t* = 200 ms, the unstable perturbation has induced a full-blown activation.

In the electronic supplementary material, we provide more detailed results as to the effect of introducing perturbations. We observe that perturbing the solution with an eigenvector associated with a positive eigenvalue results in increasing deviation between the perturbed and original solutions. Similarly, perturbation via an eigenvector associated with a negative eigenvalue typically decays to zero. Moreover, decay towards the unperturbed solution is slow when the eigenvalue is marginally negative, and fast if the eigenvalue is much smaller than zero. These observations are consistent with earlier computational results [18], where we also show related analysis employing more simplified models. Mathematical and numerical methods for computing the stability steady-state solutions have been developed [28] for a range of electrophysiological cell models.

In summary, results confirm that if the real parts of all eigenvalues are negative, as represents the typical case in healthy tissue (see the electronic supplementary material), then any small perturbation will decay locally in time. However, if the real part of an eigenvalue is positive, as occurs in disease states (i–iii), then a perturbation via the associated eigenvector leads to blow-up of the solution. We thus propose that unstable eigenmodes are one source of ectopic beats and that such modes can therefore drive arrhythmias.
